# The Effects of Growth Factors and Cytokines on Hepatic Regeneration: A Systematic Review

**DOI:** 10.7759/cureus.24539

**Published:** 2022-04-27

**Authors:** Aanal Patel, Reema Aslam, Maria Jamil, Afsana Ansari, Safeera Khan

**Affiliations:** 1 General Surgery, California Institute of Behavioral Neurosciences & Psychology, Fairfield, USA; 2 Pediatrics, California Institute of Behavioral Neurosciences & Psychology, Fairfield, USA; 3 Internal Medicine, California Institute of Behavioral Neurosciences & Psychology, Fairfield, USA; 4 Research, California Institute of Behavioral Neurosciences & Psychology, Fairfield, USA

**Keywords:** hepatic regeneration, liver transplantation, growth factor, cytokines, gdf-15, il-6, hepatocyte growth factor, hepatocyte proliferation

## Abstract

The incidence of liver disease increases throughout the years due to many lifestyle factors; thus, the only definite treatment available for chronic liver disease is a liver transplant. However, the liver has a natural ability to repair itself and regenerate its hepatic tissue from stem cells. It is hypothesized that by inducing the liver with specific growth factors and cytokines such as interleukin 6 (IL-6) compared to general growth factors like growth differentiation factor 15 (GDF-15), it can regenerate, decreasing the need for liver transplant procedures. MEDLINE, the Journal of Hepatology, and Google Scholar were used to find articles. Various studies, including epidemiological studies dated from the year 2000 and greater, were used for the introduction. The results used only randomized control trials, experimental studies, and primary articles published since 2000. This compared the results of manipulating variables to determine the effects of hepatic regeneration by either specific hepatocyte growth factors or general growth factors like GDF-15. A total of 10 collected studies showed increased levels of gene expression and function, improved gross morphology, and histological appearance of the liver when induced by cytokines and specific growth factors versus general growth factors. Overall, the hypothesis was proven. The effects of GDF-15 were not significant compared to the effects of hepatocyte-specific growth factors and cytokines like IL-6 because they have two different effects on the liver after liver injury. Future studies should investigate this topic on the human hepatic regenerative ability, plus compare the effects of general growth factors like GDF-15 and specific hepatocyte growth factors and cytokines such as IL-6 in human liver tissue.

## Introduction and background

In the past several years, end-stage liver disease (ESLD) remains an increasing cause of death. Global increases in sedentary lifestyles, overnutrition, and life expectancy predict further increased prevalence in the population with ESLD, with alcohol-induced being the primary cause in the developed countries [1]. Although many underdeveloped countries are also experiencing high levels of ESLD, the cause of the liver disease is different from that of the developed population. These countries experience high levels of ESLD due to viral causes of hepatic damage such as hepatitis C virus-induced chronic liver cirrhosis. Despite the availability of vaccines to prevent chronic liver disease (CLD) caused by viral damage, there is still an increased incidence of liver diseases such as liver failure, cirrhosis, and liver cancer [1]. This warrants an improved need for a potential treatment to reduce mortality rates in ESLD and CLDs. As there are not many chronic treatments available for liver disease patients, liver transplant primarily remains one of the definite treatments for ESLD [2].

Additionally, rather than a complete liver transplant, partial liver grafts may also be used to treat those receiving liver transplantation [3]. The process of partial liver grafts shows that within minutes to hours after liver tissue is resected, the liver begins to regenerate its healthy hepatocytes. The hepatocytes regenerated reside in the Go phase of the cell cycle (considering hepatocytes are stable cells) to enter the proliferative phase of the cell cycle. This leads to the differentiation of the liver's stem cells into the liver parenchyma's various cells that support healthy liver function. These cells involve the hepatocytes, biliary epithelial cells, sinusoidal endothelial cells, Kupffer cells, and stellate cells, for a few to name [4]. The liver graft's regenerative ability after transplantation is affected by many factors such as blood flow to the organ, portal circulation, and specifically immunologic factors [5]. Graft regeneration is done through multiple mechanisms including platelet therapies or the stimulation of cytokines such as interleukin 6 (IL-6) and tumor necrosis factor-alpha (TNF-a) [6]. However, transplantation of stem cells that induce various growth factors and cytokines can be beneficial for the liver to restore its function because it can improve damaged liver function. These stem cells can secrete high levels of cytokines and growth factors that stimulate hepatocyte regeneration. In severe liver injury, adult stem cells can also be stimulated to produce cell lines of the surrounding liver parenchyma, such as hepatic and biliary epithelial cell lineages [7]. 

Hepatocyte growth factor (HGF) is considered a mitogen for the liver as it plays a role in regenerating the liver's stem cells through a multitude of signaling pathways which can essentially promote hepatic tissue healing such as collagen deposition to create an environment that allows growth and expansion of progenitor cells [8]. More importantly, growth factors are produced by a variety of different cells in the body, and various growth factors can stimulate signaling pathways that produce secondary growth factors. For instance, after an injury to the liver occurs, the mesenchymal stem cells have been found to initially induce fibroblast growth factor 2 (FGF-2) and then have shown an overexpression of HGF through induction by the FGF-2, such that HGF provides a protective role to increase repair, restore function, and increase the regenerative capacity of the hepatocytes [2]. One such growth factor is GDF-15 (growth differentiation factor 15); this growth factor is found primarily when tissue is induced to stress, making this growth factor a key interest in studying liver graft regeneration since higher levels of GDF-15 after the liver is induced to stressful circumstances may provide benefit to the organ by possibly aiding repair of the hepatic tissue [5]. The cytokines and growth factors produce these effects through various mechanisms. However, which type of cytokine versus growth factor is more beneficial for the damaged liver must be determined to identify the accurate treatment after liver transplant in patients with ESLD and CLD.

With the concurrent rise in patients for ESLD, there is an increased need for individuals requiring liver transplants and a high need to protect existing liver damage by inducing hepatocyte regeneration to protect from further damage and liver failure [2]. However, many cytokines have been shown to provide this function; however, the roles of growth factors vary in terms of hepatocyte proliferation post-liver damage. Through reviewing and combining the knowledge from various primary literature articles, this systematic review was done to demonstrate that the liver being an organ with regenerative capacity, can selectively proliferate its hepatic tissue when induced by cytokines such as IL-6 as well as hepatocyte derived growth factors but not in the case of general growth factors such as GDF-15. 

## Review

Methods

**Figure 1:**
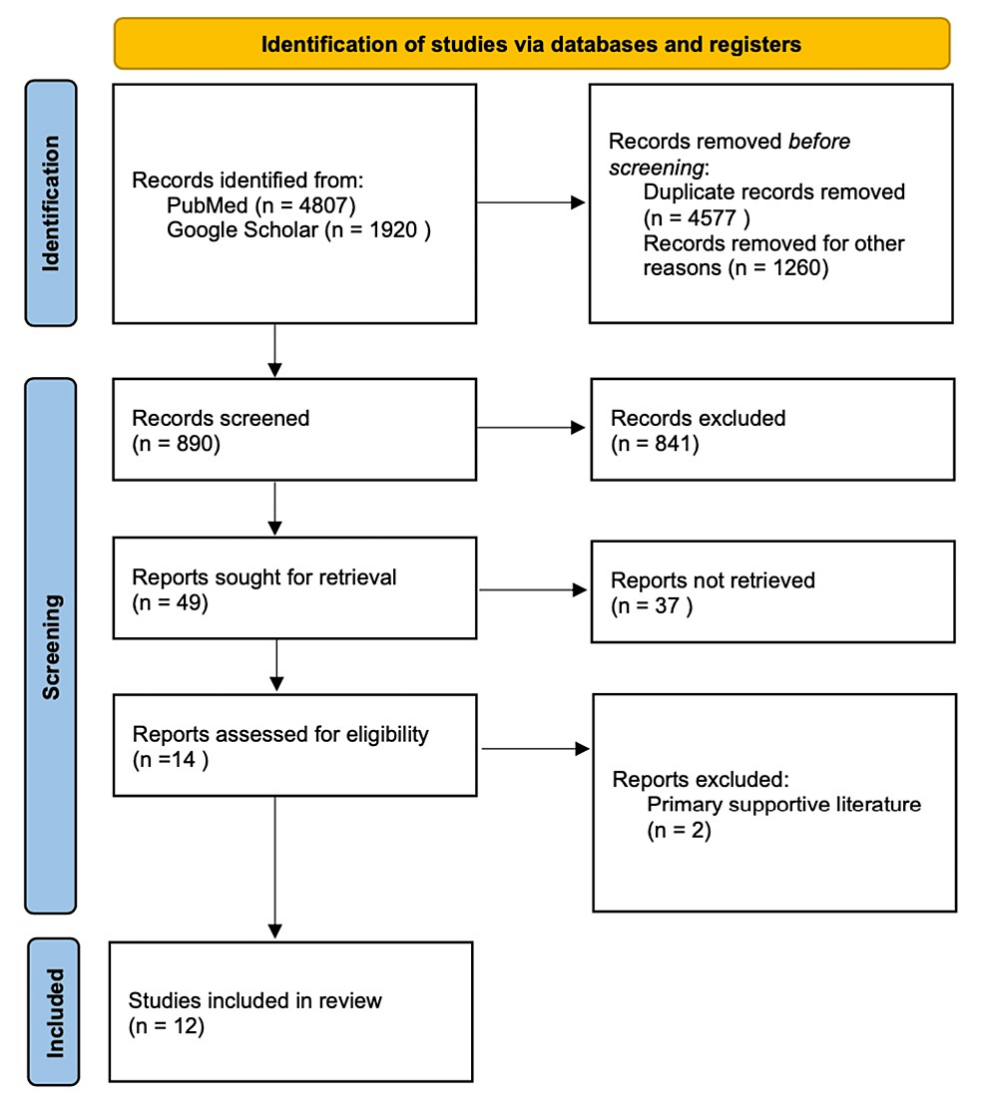
Prisma Diagram of Data Selection. PRISMA, Preferred Reporting Items for Systematic Reviews and Meta-Analyses.

We conducted a thorough literature review to find if the liver being an organ with regenerative capacity can selectively proliferate its hepatic tissue when induced by cytokines such as IL-6 and hepatocyte-derived growth factors. We followed the Preferred Reporting Items for Systematic Reviews and Meta-Analyses (PRISMA) reporting guidelines to conduct this systematic review. To narrow down the literature to evaluate this hypothesis, we developed a comprehensive search strategy to identify the supportive literature (Figure 1). 

A systematic search of PubMed and Google Scholar was conducted to identify relevant studies. A search through each primary article's references was done to determine the secondary references to further identify related studies. For the introduction of this review article, any or all articles that contained the keywords were selected and carefully examined to gather a key understanding of the information presented in each article to successfully introduce the effect of specific cytokines on the regenerative capacity of hepatic tissue. Both primary and secondary review studies and epidemiological studies were used to supplement the introduction, whereas the results consisted solely of primary articles. The articles were chosen by reviewing the title and reading through the abstract to identify that the study's main goal and its results supported the hypothesis to make sure the study was qualified.

Furthermore, the introduction of the articles provided beneficial information regarding the population and type of the study; this helped toward supporting the hypothesis as a population of humans was preferred; although many were solely experimental based on mice, these studies were not excluded because their results supported the hypothesis as a general statement toward hepatic regeneration rather than specifically addressing the human hepatic regenerative capacity. The following keywords - liver regeneration, GDF-15, hepatocyte growth factor, chronic liver disease, cytokines, partial hepatectomy-aided in the process to narrow down as many eligible studies as possible. Additionally, the search terms were combined further to gather relevant studies. The studies were selected by whether or not they qualify through an inclusion and exclusion process. The MeSH keywords were used to develop group search terms combined with the keywords that provided the total amount of eligible studies in the PubMed search engine.

Inclusion Criteria

The published papers to be included were limited to the studies published in the year 2000 and later, larger study population, and more trials to increase the individual study's quality. The majority of the articles were primary articles to support the hypothesis and experimental procedures that studied the therapeutic effects of specific cytokines in inducing hepatic regeneration.

Exclusion Criteria

Studies excluded were review articles along with those of older dates of publication. Additionally, articles in books and document formation were excluded from PubMed's initial search. Many articles were excluded as a result of title screening; the remaining articles were removed after a complete abstract and article read of the studies selected after the title screen exclusion.

Quality Check

A quality check of the total articles selected was done as applicable to each study type using the AMSTAR checklist; a total score of five or greater resulted in the study being included as part of this review. For any clinical trials, the Cochrane Bias Assessment tool was used; for observational studies and non-randomized control trials, the New Castle Ottawa tool was used.

Results

Overall search results from all databases used were 6,727 articles; duplicates were removed, resulting in 890 articles. These articles were reviewed using inclusion and exclusion criteria to narrow down eligible studies further. The overall number of studies included after the complete search strategy, inclusion and exclusion criteria, and quality check are demonstrated through the PRISMA flow chart (Figure 1). A total of 14 articles were screened for quality checks that qualified at level five and above (Table 1). 

**Table 1:** Evidence Table IL-6, interleukin 6; GDF-15, growth differentiation factor 15; TNF-a, tumor necrosis factor alpha; RCT, randomized controlled trial; MSCs, mesenchymal stem cells; LDLT, living donor liver transplantation; HGF, hepatocyte growth factor; LPS, lipopolysaccharide.

First Author	Date of Publication	Study Design	Level of Evidence	Study Population	Therapy or Exposure	Outcome/Results
Banas, A [2]	October 2008	Animal experimental study	III	Mice	Adipose tissue mesenchymal cells	The therapeutic effects on the liver of inducing mice models with adipose tissue MSCs
Hsiao, EC [6]	May 2000	Animal laboratory study	I	Mice	GDF-15	Mice induced with GDF-15 did not experience any extra hepatocyte proliferation than wild-type mice
Norris, CA [8]	April 2014	Animal laboratory study	I	Rats	IL-6 response	Pure Kupffer cell extracts had a reduced IL-6 response compared to hepatocyte cultures
Choi, JS [9]	April 2019	Experimental study	III	Human adipose mesenchymal stem cells	Cytokines	Transplanting human adipose mesenchymal stem cells into damaged liver reduced fibrosis and promoted liver regeneration
Takahashi, K [10]	November 2019	Review article	II	Liver resection and regeneration	Platelet therapy	Using platelet therapy to assess the regeneration of liver tissue after post-hepatectomy liver failure
Pintilie, DG [11]	August 2010	Animal laboratory study	II	Mice hepatic stellate and oval cells	L-cysteine	Induction of L-cysteine showed a negative effect on activation of hepatic stellate and oval cells and reduced the liver's expression of alpha-fetoprotein
Riehle, KJ [12]	January 2011	Review article	III	Mammalian liver	Progenitor therapy	Challenges between various therapies in inducing liver proliferation
Streetz, K [13]	December 2003	Animal laboratory study	III	Mice	IL-6	IL-6 was protective in liver damage due to liver disease in mice supplemented with the cytokine
Chae, MS [14]	April 2018	RCT	IV	Humans	IL-6 and TNF-a response	LDLT patients supplemented with IL-6 and TNF-a were successfully able to undergo liver regeneration
Limdi JK [15]	January 2003	Review article	I	Humans	Liver disease	The effects of various liver function tests and markers after exposure to different types of liver disease
Li, M [16]	June 2018	Animal laboratory study	III	Mice	GDF-15	Mice exposed to GDF-15 experienced lower levels of inflammation and reduced LPS-induced liver damage
Ishikawa, T [17]	November 2011	Animal laboratory study	III	Mice	Growth factor HGF/C-met	Mice lacking c-met were not able to regenerate their liver and developed severe liver atrophy

After the quality check, two articles were removed as primary supportive literature resulting in 12 total articles. However, these articles were still used as secondary supporting literature due to important data that answered the question as to whether antiepileptic drugs can play a beneficial role in preventing cerebral ischemia for surgical patients prone to blood loss due to in-surgical hemorrhagic shock. For systematic reviews and meta-analysis studies, the AMSTAR checklist was used; a total score of five or greater resulted in the study being included as part of this review. For any clinical trials, the Cochrane Bias Assessment tool was used; for observational studies and non-randomized control trials, the New Castle Ottawa tool was used. The articles remaining after this quality assessment was used as the primary supportive literature. The overall number of studies included after the complete search strategy, inclusion and exclusion criteria, as well as a quality check are demonstrated through the PRISMA flow chart (Figure 1).

There was a total of six animal laboratory studies, one randomized control trial, two experimental studies, and one review article used that supported the beneficial role that cytokines and specific growth factors played in allowing the proliferation of liver tissue and supporting the regeneration of the cells in the hepatic parenchyma.

Discussion

Of the total studies assessed, nine were relevant to supporting the hypothesis because they successfully studied the overall effects of cytokines' gene expression and compared their effects to the effects of growth factors after liver injury [8]. They also monitored the effects of hepatic regeneration after induction with external general growth factors, specific hepatic growth factors, and cytokines [10,12]. 

The hypothesis supported one study's findings stating that hepatic growth factors have a stimulatory role in the regeneration of hepatic cells and inducing angiogenesis in the hepatic parenchyma [13]. Angiogenesis can help clear debris from initial hepatic damage by allowing increased blood flow and bringing in more inflammatory cells to the area of damage, whereas cytokine induction not only has a stimulatory role but has an additional function of downregulating fibrotic changes in hepatic tissue after insult to prevent fibrosis and thus inducing a larger effect on hepatic regenerative capacity [13]. Some limitations found in this study were that it was conducted on adipocyte-derived stem cells rather than hepatocyte stem cells. This study could not also test on the human liver since it was an experimental animal study generalized toward mice liver tissue, which may or may not apply to human hepatocyte regeneration. Thus, although supporting the overall hypothesis with its identification of the functions of general, HGFs and cytokines as well as making comparisons between the two, the study results were applied to mice models and were unable to compare to humans; the study also could not assure that this information was applicable and accurate toward human hepatic tissue in exposure to growth factors compared to cytokines. 

The study discusses cytokines, and there is no comparison to how their effects differ from growth factors [12]. However, this study supports the idea of cytokines' beneficial role in liver damage by considering that cytokines are the predominant cause of increased liver regeneration following damage to hepatic tissue. Even though this study fails to consider the role of cytokines in comparison to growth factors in terms of hepatic regeneration, the results support the hypothesis that cytokines do have a key role (although minor) to play in hepatic regeneration, as seen in the liver grafts from postoperative patients [12]. Human liver from patients with ESLD was used by performing a partial hepatectomy rather than experimental mice liver, which supports the audience targeted by the hypothesis. A generalization of IL-6 and TNF-a's role was made toward patients with ESLD preoperative and postoperative hepatic regenerative capacity [12,18]. Limitations include not measuring cytokines in liver graft tissue, thus being unable to quantify the increase or decrease in cytokines. Despite the limitations, IL-6, induced by HGF [9], and TNF-a were correctly identified to play a role in liver graft regeneration [10,19].

Additionally, the signaling pathways involved in inducing the proliferation of hepatocyte stem cells and developing an environment for differentiation and growth of the hepatic tissue were effectively studied. This experimental study was conducted on wild-type mice and control mice; the wild type lacked the c-met signaling pathway, and the control had the pathway to allow proliferation. The generalization of the results was primarily for mice models and had limited support for the human liver. The results suggested that the pathway was primarily stimulated by cytokines and growth factors, thus creating a downstream effect on regenerating hepatic tissue after a toxic injury to the liver [15]. This study does support the theory that cytokines play a large role in hepatic regeneration. However, it fails to compare the differences between cytokines' effects and how their pathways differ from the effects of growth factors, thus not entirely supporting the hypothesis that growth factors have a larger role in hepatic regeneration than cytokines. Future implications should involve comparing the beneficial effects of cytokines to growth factors rather than solely cytokines and studying the signaling pathways in human liver tissue.

The effects of GDF-15 and cytokines on hepatocyte regenerative capacity are studied, finding that GDF-15 plays a preventative role in further liver damage by inflammatory cells after injury [14]. This study accurately supports the hypothesis through its finding that GDF-15 lowers the expression of pro-inflammatory cytokines, thereby reducing damage (characterized by the serum aspartate aminotransferase/aspartate aminotransferase [AST/ALT] levels before and after GDF-15 exposure) in hepatic tissue after insult rather than inducing proliferation of hepatic stem cells or aiding in regeneration [14]. However, similar to another study, it fails to compare hepatic tissue induced to GDF-15 alone versus induced along with cytokines, making the general assumption that GDF-15 leads to cytokine release [14,15]. Like other studies, this study used mice models; thus, the results are a generalization of all animal liver tissues. It would be beneficial if future studies could involve monitoring the effects of GDF-15 on the regeneration of human liver tissue. 

The effects of HGF and its relation to IL-6 are analyzed, finding that HGF leads to increased gene expression of IL-6, higher levels of both were found in mice with liver damage, and increased levels were related to increased mice survival [8]. This experimental study uses mice models; further recommendation of following IL-6 levels in human liver survival is recommended. This study does support the hypothesis as to its states the HGF, and IL-6 cytokine levels are partially related, the beneficial effect of HGF is in turn due to the downstream effect of increased IL-6 and other pro-inflammatory cytokines that prevent liver damage and stimulate stem cell proliferation to induce hepatic regeneration [8]. Similarly, the effects of removing IL-6 function were tested - IL-6 gene pathways were deleted in knockout mice hepatocytes versus control mice hepatocytes, and the liver was induced with damaging toxic substances [11]. As the disease progressed, IL-6 was activated at higher levels which essentially provided protective effects on the liver. The hepatic tissue of the knockout mice was noticed to have rapidly progressed to fibrotic tissue compared to the control, thus demonstrating that the presence of IL-6 cytokine provides a protective and proliferative role in hepatic stem cells, increasing their ability to regenerate and repair hepatic tissue [11]. 

Furthermore, hepatocytes were exposed to damaging chemicals such as CCl4 and ethanol to destroy the hepatic tissue and observe the repair process [16]. The ethanol induction caused acute alcoholic hepatitis in the mice's liver; this was done by altering the pathways metabolizing lipids. The acute alcoholic hepatitis caused by ethanol leads to visible damage to liver surface contour and gross appearance [16]. They discovered an increased expression of GDF-15 mRNA levels after hepatic injury. These GDF-15 levels were instantly and drastically elevated in mice experiencing an ethanol-induced hepatic injury, primarily in the liver tissue [5]. However, these levels were not related to chronic damage and repair; instead, they solely represent an acute inflammatory response. The null mice in whom the GDF-15 production was experimentally inhibited (done through a laboratory-induced deletion in the gene responsible for expressing GDF-15) still experience the same hepatic repair and regeneration levels as the mice in which the GDF-15 production pathway was intact [16]. The extent of liver damage and necrosis of the damaged hepatocytes after injury was measured by ALT/AST levels-cytosolic enzymes found at the highest concentration in the liver and leaked out into the serum following cellular damage [13]. These levels were highest near the centrilobular area, which is the primary area affected by the toxic insult [16]. The higher the expression of GDF-15 mRNA, the lower the AST/ALT levels suggesting that GDF-15 played a role in reducing damage by reducing the acute inflammatory response responsible for additional hepatic damage rather than regenerating the hepatic and restoring function [16]. 

A further investigation of the effects of removing cytokine stores was done; a compound characterized by L-cysteine was found to shut down cytokines such as IL-6 and TNF-a proliferation, leading to loss of cytokine stores [9,17]. The more L-cysteine that was fed to the mice, the lower the levels of hepatic regeneration. These lower hepatic mass levels were due to reduced stellate cell proliferation and reduced oval cell proliferation and differentiation [9]. Stellate cells and oval cells are the predominant stem cell types that hepatic regeneration begins with; the loss of cytokines stores from the L-cysteine diet leads to reduced function of these cells, essentially leading to decreased ability of hepatic repair [9]. This study used mice models, generalizing that these effects would also be similar in human liver tissue. Further studies should identify whether or not the L-cysteine diet is safe in humans to explore the similarity of these effects in humans compared to mice. 

Limitations

Future studies should use human patients for their experimental variables rather than mice; this would provide better results for how the liver would regenerate after partial resection in patients with ESLD or CLD and post-transplant patients. Additionally, a larger pool of subjects would help provide more accurate data by correcting patients' loss through illness, mortality, or simple exclusion. Thus, a larger group of subjects would lower the statistical standard of error, providing more accurate data. Future studies should study both the effects of growth factors and cytokines and compare their effects on gene expression, mitogenesis, stem cell differentiation, gross effects on the liver, and survival potential in patients to provide better data on which is more efficient in inducing hepatic regeneration. Also, the survival rates through gross liver examination and gene expression studies compared with each of the induction variables should be analyzed. This ensures accurate evidence of the effects of growth factors versus cytokines on the strength of their ability to induce hepatic proliferation after damage to liver tissue.

## Conclusions

Through animal and mice studies, it was concluded that GDF-15 and general growth factors provide a protective effect by downregulating more inflammatory damage in hepatic cells as determined through the various experiments conducted on mice livers, and specific growth factors such as HGF and cytokines such as IL-6 have an increased effect on the growth of the hepatic tissue by inducing the hepatocyte stem cells to regenerate the liver parenchyma.
